# Advancing Hair Regrowth Assessment: Development and Standardization of Methods for Evaluating New Hair Growth, Hair Keratin Levels, and Scalp Health in Androgenetic Alopecia Patients

**DOI:** 10.7759/cureus.79859

**Published:** 2025-02-28

**Authors:** Maheshvari N Patel, Nayan Patel, Apeksha Merja

**Affiliations:** 1 Clinical Research, NovoBliss Research Private Limited, Ahmedabad, IND; 2 Pharmacology, Swaminarayan University, Ahmedabad, IND; 3 Dermatology, NovoBliss Research Private Limited, Ahmedabad, IND

**Keywords:** androgenetic alopecia, hair keratin, hair regrowth, inter-evaluator variability, new hair growth

## Abstract

Introduction

Androgenetic alopecia (AGA) is a prevalent and progressive hair disorder affecting both men and women. It is characterized by the miniaturization of hair follicles, reduced hair density, and alterations in scalp conditions. Accurate assessment of hair growth parameters is crucial for understanding the progression of AGA and evaluating the efficacy of treatment interventions. This study aimed to develop and validate robust methodologies for assessing critical endpoints related to hair growth and scalp health. These standardized methods provide a reliable framework for future efficacy studies targeting AGA and similar hair disorders.

Methods

This method development and standardization study utilized an observational, non-interventional, open-label design and included 25 subjects diagnosed with AGA. Non-invasive techniques were employed to evaluate various hair parameters, including hair regrowth (vellus hair count), hair density, thickness, and total hair count, using the CASLite Nova phototrichogram software (Catseye Systems & Solutions Pvt Ltd, Navi Mumbai, India) and Image-Pro software (Media Cybernetics, Inc., Rockville, MD, USA) for precise analysis. Hair length was assessed using a calibrated ruler, while hair tensile strength was measured with the Testronix tensile hair tester (TX TST C, Testronix, Inc., Manila, PHL) instrument. Additional parameters, such as quantitative hair keratin levels and scalp keratin levels, were analyzed to provide an understanding of hair health. Baldness severity was evaluated using the Norwood-Hamilton scoring system for male participants and the Ludwig scoring system for female participants. Furthermore, a detailed visual hair distribution analysis was conducted. To minimize inter-evaluator variability, standardized training sessions were conducted, ensuring consistency across assessments. The reliability of the scoring systems was confirmed using the Fleiss Multirater Kappa test. This study received ACEAS Independent Ethics Committee (EC) approval on July 10, 2024.

Results

The vellus hair count, indicative of new hair growth, was found to be higher in AGA-affected areas, averaging 12.4 compared to 3.92 in the standardized area. Terminal hair counts were lower in AGA-affected areas, with 9.88 compared to 21.84 in the standardized area. Hair density (count/cm²) and thickness (µm) were significantly less in AGA-affected areas, with averages of 209.84 (count/cm²) and 6.64 µm, compared to 246.12 (count/cm²) and 9.16 µm in the standardized areas (p < 0.0001). Tensile strength was measured at an average of 344.14 MPa across samples. The Fleiss Multirater Kappa analysis showed high agreement between evaluators for Norwood-Hamilton (0.894) and Ludwig scores (0.957).

Conclusion

This standardization and validation study underscores the importance of developing and utilizing standardized methodologies in clinical research, particularly in hair and scalp health studies. The implementation of these accurate and reproducible techniques will enhance the reliability of efficacy data for both current and future AGA treatments while establishing a benchmark for advancing research in hair restoration and scalp health. These methodologies provide a reliable and scientifically grounded approach to studying hair regrowth, paving the way for evidence-based treatments that can address the diverse needs of individuals with AGA and other hair-related disorders.

## Introduction

Androgenetic alopecia (AGA), also known as male or female pattern baldness, is the most common form of hair loss affecting both men and women worldwide [1]. It is characterized by progressive thinning of the hair, primarily in the frontal and vertex regions in males, and a diffuse thinning over the crown in females. AGA is a hereditary condition that affects up to 50% of men and women by the age of 50, making it a significant cosmetic and psychological concern for many individuals [2]. While the exact pathophysiology of AGA remains partially understood, it is primarily driven by genetic predisposition and hormonal factors. The primary culprit is dihydrotestosterone (DHT), a derivative of testosterone, which binds to androgen receptors in the hair follicles, leading to follicular miniaturization. This miniaturization results in the production of progressively finer and shorter hair shafts until the follicles become inactive, causing visible thinning and baldness [3]. The psychological impact of AGA can be profound, often leading to lowered self-esteem, anxiety, and depression [4].

Currently, several treatment options are available to manage AGA, aimed at either slowing down hair loss or promoting hair regrowth. Among the pharmacological treatments, topical minoxidil and oral finasteride are the only Food and Drug Administration (FDA)-approved medications for AGA. Minoxidil, a topical vasodilator, is believed to enhance hair growth by increasing blood flow to the hair follicles and prolonging the anagen (growth) phase of the hair cycle [5,6]. Finasteride, an oral 5-alpha-reductase inhibitor, reduces DHT levels, thereby preventing follicular miniaturization. However, both treatments have limitations, including variable efficacy, the need for prolonged use, and potential side effects such as scalp irritation or sexual dysfunction [7]. In addition to these conventional treatments, there is growing interest in the use of natural and alternative therapies for AGA, which is traditionally used in Ayurveda to promote hair growth [8]. Herbal oil is believed to have anti-inflammatory and vasodilatory properties that may contribute to hair regrowth. Furthermore, oral supplements containing omega-3 fatty acids and biotin have gained popularity due to their potential benefits for hair health, although robust clinical evidence supporting their efficacy in AGA is still limited [9,10].

Given the broad spectrum of available treatments, there is a pressing need for standardized methods to assess the efficacy of these interventions objectively. Hair growth studies often focus on parameters such as hair density, thickness, length, and tensile strength. However, one of the critical gaps in the literature is the lack of standardized methods for measuring the regrowth of new hair, particularly in AGA-affected areas. While several validated instruments and processes exist, such as phototrichogram analysis and dermatological assessments, most studies primarily focus on the non-affected or less-affected scalp regions. This gap creates a challenge in comparing the effectiveness of different treatments, as the evaluation of hair regrowth in AGA-affected regions could provide more meaningful insights into treatment efficacy.

The present study aims to address this gap by validating a comprehensive set of methods for assessing multiple hair growth parameters, including new hair regrowth, in both AGA-affected regions and standard scalp areas. The study employed a multi-faceted approach to evaluate hair growth, thickness, density, and tensile strength using advanced tools like CASLite Nova (Catseye Systems & Solutions Pvt Ltd, Navi Mumbai, India) for phototrichogram analysis and Testronix (TX TST C, Testronix, Inc., Manila, PHL) for tensile strength measurement. The assessment of hair length was carried out using a standardized ruler method, while digital photographs were taken to provide visual documentation of the changes in the head crown area over time. Additionally, dermatological evaluation using the Norwood-Hamilton classification for males and the Ludwig classification for females provided a standardized approach to assess the severity of alopecia. Moreover, the quantitative measurement of scalp keratin using the Bradford assay offered a unique perspective on protein content changes in the hair, which may correlate with hair health and strength.

The rationale for this study is anchored in the need for a comprehensive and standardized assessment protocol that can accurately evaluate the efficacy of various hair treatments in promoting hair regrowth, especially in AGA-affected areas. By standardizing the methods for measuring multiple hair growth parameters, this study seeks to provide a robust framework that can be adopted in future clinical trials and studies investigating the effectiveness of new and existing hair treatments. Such standardized approaches will not only improve the reliability and comparability of study outcomes but also help in the development of more targeted and effective treatment strategies for managing AGA.

## Materials and methods

Ethical conduct of the study

This study was conducted according to the ethical principles outlined in the Declaration of Helsinki, the International Council for Harmonization (ICH) guidelines, and the Good Clinical Practice (GCP) guidelines to ensure the protection of the subjects' rights, safety, and well-being.

The study protocol (version 1.0, dated July 4, 2024), case report form (version 1.0, dated July 25, 2024), investigator's undertaking, CV, medical research council (MRC) of the principal investigator, and list of participating investigators were reviewed and approved by the ACEAS Independent Ethics Committee before study conduct on July 10, 2024. Furthermore, this study was registered with ClinicalTrials.gov (NCT06802237).

All participants provided written informed consent before any study-related procedures were performed. The participants were fully informed about the study's objectives, methodologies, potential risks, and benefits. Throughout the study, the confidentiality of participants' data was maintained, and their identities were protected. Any deviations from the protocol were documented and reported in accordance with regulatory requirements.

Study design

This study followed an observational, non-interventional, open-label design aimed at developing a standardized methodology for assessing hair regrowth, number of new hairs, hair length, hair strength, scalp and hair keratin analysis, quantitative scoring of baldness and its inter-variability, and global head crown photographs in patients with AGA. The study involved 25 subjects with mild to moderate AGA, aged 18-55 years. The objective was to develop a comprehensive methodology that incorporated various clinical, instrumental, and visual assessments to evaluate hair growth and scalp health in both AGA-affected and standardized scalp regions.

Method of standardization and methodology

Several instrumental and visual evaluations were performed to gather comprehensive data.

Hair Regrowth Evaluation

**Figure 1:**
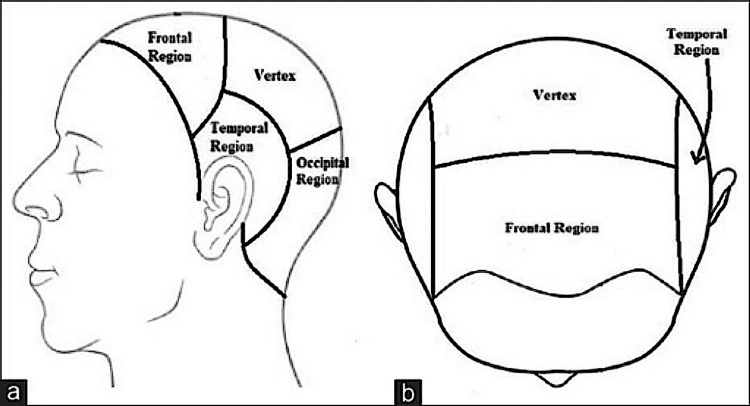
Scalp region: (a) side view; (b) top view Image created by the authors.

Standardized site marking ensures consistent hair regrowth assessment. Hair regrowth assessments from the AGA-affected area (in males) utilized standardized site marking with a calibrated tape and skin marker. A marking was placed near the eye area, with a perpendicular line drawn on the bald scalp. Hair regrowth (in females) from the AGA-affected area was assessed from the standardized area at the vertex region of the head scalp, i.e., 30 cm towards the head from the nose tip. Images were captured using CASLite Nova to focus on the target area, evaluating the vellus hair and terminal hair, ensuring accuracy and repeatability in evaluations (Figure 1).

New Hair Count

**Figure 2:**
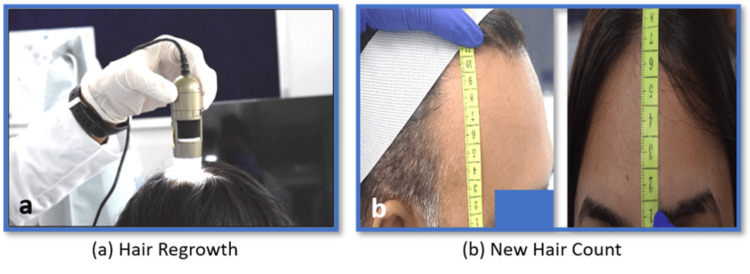
(a) Hair growth assessment; (b) new hair count

Hair counts in AGA-affected areas were analyzed using the CASLite Nova phototrichogram, and additional new hair counts were evaluated through Image-Pro software (Media Cybernetics, Inc., Rockville, MD, USA) for a comprehensive assessment (Figure 2).

Keratin Level Assessment

Quantitative hair keratin analysis: Hair keratin levels were precisely measured using the Bradford assay, ensuring an accurate evaluation of keratin concentration in hair strands.

**Figure 3:**
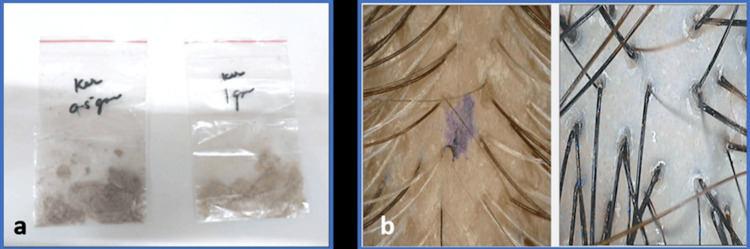
(a) Hair keratin assessment; (b) scalp keratin assessment

Quantitative scalp keratin analysis: Scalp keratin levels were evaluated using a phototrichogram with CASLiteNova, which captured detailed scalp images to analyze optical density and other keratin-related features. This technology enabled consistent and precise measurement, providing critical insights into scalp health (Figure 3) [11].

Hair Length Assessment

Hair length was measured using a calibrated ruler and skin marker. A designated spot was marked 15 cm from the nose tip toward the scalp vertex, and hair strands were extended downward for measurement. This precise method ensured consistency in data collection for clinical studies.

Hair Tensile Strength

**Figure 4:**
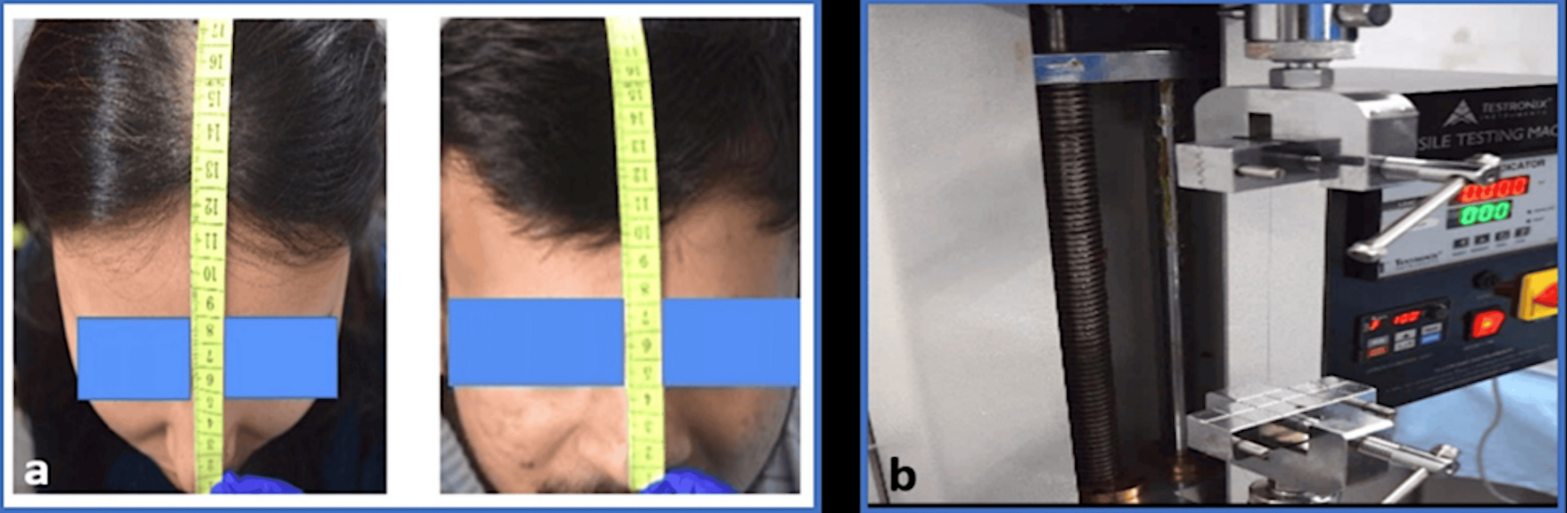
(a) Hair length assessment; (b) tensile strength assessment

Hair tensile strength was assessed using the Testronix tensile hair tester, where a single hair strand sample was fixed and a pulling force was applied until breakage. Tensile strength was calculated based on the force and the hair's cross-sectional area, with results expressed in megapascals (MPa). This method provided precise and uniform tensile strength assessment (Figure 4) [12].

Hair Distribution Analysis

**Figure 5:**
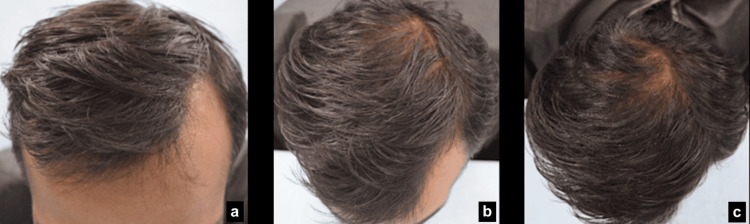
Global head crown imaging at different angles (a-c)

Standardized imaging of the global head crown at multiple angles (0°, 45°, 90°) provided a clear view of hair distribution and alopecia severity. A tripod standardized marking ensured consistent camera positioning, while twin flashes offered uniform lighting for reliable assessments (Figure 5).

Inter-evaluator variability

To ensure consistency in hair loss assessment, inter-evaluator variability was examined by comparing the scores assigned by seven independent evaluators across the 25 subjects. The evaluations included grading hair loss using the Norwood-Hamilton classification for males and the Ludwig classification for females. The Fleiss' Multirater Kappa was used to quantify the agreement between evaluators, demonstrating the reliability of the scoring methodologies and ensuring that results were reproducible.

In summary, this study successfully developed a comprehensive methodology for assessing hair growth and scalp health in AGA patients using a combination of instrumental, clinical, and visual techniques. The study design incorporated rigorous evaluator training and reliability assessments, ensuring the precision and reproducibility of the measurements.

Sample size determination

A total of 25 subjects were enrolled using the convenience sampling method; participants were selected based on accessibility and feasibility. This approach was suitable for the study's objective, which was to standardize and validate the assessment methods and inter-observer reliability, and no therapeutic interventions were used in this study. The selected sample size was sufficient for methodological validation, ensuring the reliability and consistency of the assessment techniques. All 25 participants successfully completed the study and were included in the final data analysis.

Statistical analysis

IBM SPSS Statistics for Windows, Version 29 (Released 2023; IBM Corp., Armonk, New York), was used to analyze the study data. Continuous variables such as hair regrowth, hair count, hair length, thickness, and tensile strength were described using descriptive statistics, including mean, standard deviation, median, minimum, and maximum values. Categorical variables, including hair loss grades based on the Norwood-Hamilton and Ludwig scales, were expressed as frequencies and percentages. Where appropriate, graphical presentations of the data were also generated.

The Fleiss Multirater Kappa was utilized to assess the reliability and consistency of the evaluations conducted by different raters, i.e., Norwood-Hamilton scoring and Ludwig scoring. This statistical measure quantifies the level of agreement among multiple raters when classifying items into categories. Fleiss Kappa values range from -1 to 1, where a value of 1 indicates perfect agreement; a value of 0 indicates no agreement beyond chance; a value less than 0 indicates less agreement than expected by chance. High Fleiss Kappa values indicate that the assessment methods are reliable and reproducible across different raters.

Study disposition

The study commenced on July 26, 2024, and all 25 subjects were enrolled, completed, and included in the final statistical analysis. None of the subjects withdrew from the study. All study procedures were completed in accordance with the pre-approved protocol, with no significant deviations reported.

## Results

Evaluation of vellus hair count

The primary endpoint of this study focused on evaluating vellus hair count to identify new hair growth across different locations on the same scalp: one from the AGA-affected area (site 1) and another from a standardized, unaffected area (site 2). Using the standardized site marking and CASLite Nova instrument, hair regrowth in the AGA-affected area (site 1) was characterized by an average vellus hair count of 12.40, significantly higher than the 3.92 observed in the standardized area (site 2). This difference, supported by a highly significant p-value of less than 0.001, underscores the distinct variation in hair characteristics between these two regions. Furthermore, the AGA-affected area demonstrated a lower terminal hair count, averaging 9.88 hairs, in contrast to the 21.84 terminal hairs in the standardized area.

**Figure 6:**
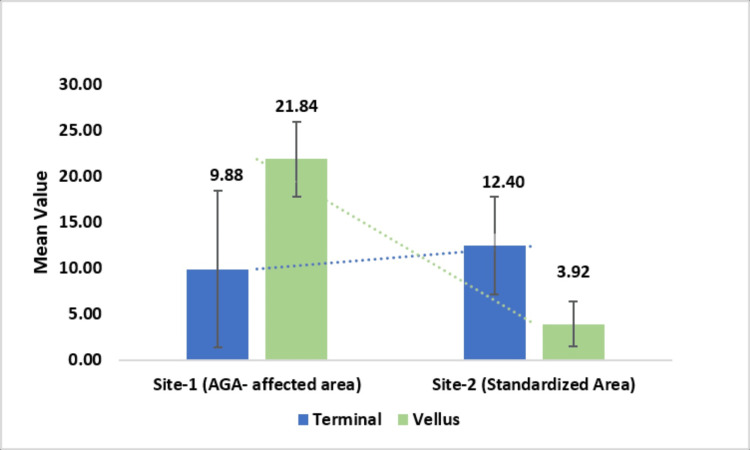
Terminal hair and vellus hair count of the AGA-affected area and standardized area AGA: androgenetic alopecia

These results highlight the importance of assessing multiple scalp locations using a standardized method to accurately distinguish between affected and unaffected regions. This provides a reliable basis for evaluating the efficacy of future treatments for AGA (Figure 6).

Evaluation of hair density and thickness

Hair density and thickness assessments were integral secondary endpoints in this study, conducted using the CASLite Nova instrument and a standardized methodology to ensure accuracy and reproducibility. Hair density was measured at 209.84 hairs per cm² in the AGA-affected area compared to 246.12 hairs per cm² in the standardized area, with the difference being statistically significant (p < 0.0001). Similarly, hair thickness was evaluated, showing an average of 6.64 µm in the AGA-affected area and 9.16 µm in the standardized area, with a statistically significant difference (p < 0.01).

**Figure 7:**
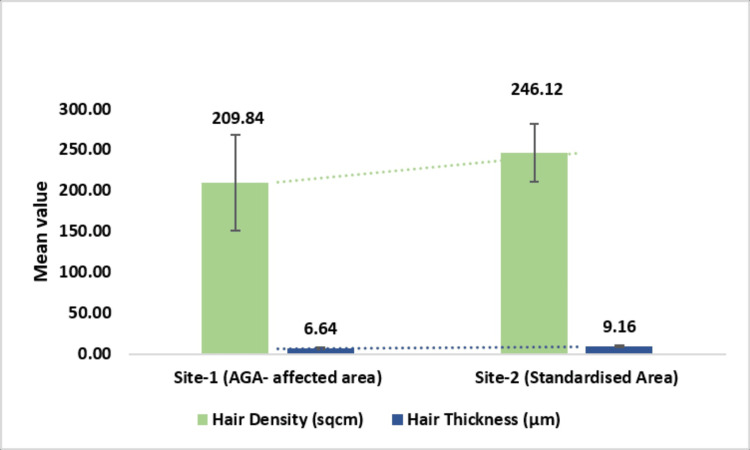
Hair density (cm²) and hair thickness (µm) assessed by CASLite Nova AGA: androgenetic alopecia

The meticulous application of this focused methodology ensured consistent and reliable data collection, providing robust insights into hair characteristics across different scalp regions, which can be leveraged in future efficacy studies for hair loss treatments (Figure 7).

Evaluation of hair keratin and scalp keratin

Hair keratin levels were quantitatively assessed using the Bradford assay method, a well-established and reliable biochemical technique designed for precise analysis of structural proteins. For this assessment, 0.05 g of hair was meticulously sampled, ensuring accuracy in the measurement process. The Bradford assay method enabled the precise quantification of keratin content, offering critical insights into hair protein composition. This robust methodology played a pivotal role in evaluating hair keratin levels [13].

The condition of the scalp, particularly keratin content, was assessed using the CASLite Nova instrument, employing a standardized methodology to ensure precise and reproducible measurements. The analysis revealed that 100% of the evaluated scalp zones exhibited measurable levels of keratin, indicating overall good scalp health across both AGA-affected and standardized areas. By leveraging this robust methodology, the assessment provided a consistent and reliable approach to evaluating scalp conditions, offering critical insights into the impact and efficacy of hair care products on scalp health.

Evaluation of hair length

Hair length was evaluated using two distinct methodologies, each based on specific characteristics of the scalp regions. In the AGA-affected area (site 1), the phototrichogram method was employed, providing precise measurements of hair lengths. The average hair length recorded was 434.88 µm. Conversely, in the standardized area (site 2), hair length was assessed using a calibrated measuring tape, revealing an average length of 20.81 cm.

By employing these separate, methodologically appropriate approaches, the study ensured accurate and standardized assessments of hair growth potential and length across different scalp regions, establishing a reliable framework for future research.

Inter-evaluator variability

To ensure reliability, the study also explored inter-evaluator variability in hair loss scoring. Scalp examinations were conducted on male subjects using the Norwood-Hamilton scale, and female subjects were evaluated using the Ludwig scale after training by a dermatologist. The agreement among evaluators was quantified, revealing a Fleiss Multirater Kappa of 0.894 (p < 0.001) for male subjects and 0.957 (p < 0.001) for female subjects. This methodological rigor ensures consistent and reproducible evaluations, providing a reliable foundation for future studies on hair loss and treatment efficacy.

Lastly, hair tensile strength was evaluated using a method based on the formula T = F/A, where T is the tensile strength, F is the applied force, and A is the cross-sectional area of the hair strand. The average force required to break hair strands was measured at 0.05 g, with an average cross-sectional area resulting in a tensile strength of 344.14 MPa. This methodical approach ensured accurate and reproducible measurements, providing a reliable quantitative baseline for assessing hair resilience and structural integrity under varying conditions. Such detailed evaluation is critical for understanding hair behavior and its response to treatments or external stressors.

In summary, this study established detailed and standardized methodologies for evaluating key parameters, including hair regrowth, density, thickness, length, scalp keratin levels, hair keratin content, and tensile strength. These methods were rigorously validated through statistically significant findings, ensuring their reliability and reproducibility. Designed for application in future research, these methodologies provide robust endpoints to assess the efficacy of hair care products in managing AGA. By focusing on the development of consistent and objective evaluation techniques, this study lays a strong foundation for future efficacy-based research without making claims about altering the parameters themselves.

## Discussion

This study aimed to establish robust and standardized methodologies for the comprehensive assessment of various hair and scalp parameters, including hair regrowth, density, thickness, length, scalp keratin levels, hair keratin content, and tensile strength. These methods were specifically designed to ensure precision, consistency, and reproducibility, providing reliable tools for future efficacy studies targeting AGA [14].

Hair regrowth was assessed using a dual-location methodology to capture the distinct characteristics of the AGA-affected and standardized areas on the scalp. By employing the CASLite Nova instrument and standardized site marking, vellus hair count and terminal hair count were precisely measured, revealing significant differences between the two regions. This standardized approach allowed for a clear distinction between the affected and unaffected areas, providing a reliable basis for future treatment assessments.

Hair density and thickness were evaluated as secondary endpoints using the CASLite Nova. The methodology ensured meticulous and reproducible measurements, with significant variations observed between the AGA-affected and standardized areas. These findings underscore the effectiveness of the methodological framework in providing detailed insights into hair characteristics [15].

Hair length was assessed using two distinct methodologies tailored to the characteristics of the targeted scalp regions. The phototrichogram method was employed in the AGA-affected area to measure hair lengths, while a calibrated measuring tape was used in the standardized area to measure longer hair strands. These complementary techniques ensured accurate and consistent measurements, emphasizing the importance of method-specific approaches in capturing hair growth data.

Scalp keratin levels were quantified using the CASLite Nova instrument, which provided measurable insights into the condition of both AGA-affected and standardized scalp areas. The uniform detection of keratin across all scalp zones evaluated confirmed the instrument’s reliability in assessing scalp health, a critical parameter in determining the efficacy of hair care interventions.

Hair keratin content was assessed using the Bradford assay method, a highly precise biochemical technique. This methodology involved the analysis of hair samples, allowing for the accurate quantification of keratin levels. The results provided valuable insights into the protein composition of hair, contributing to a more comprehensive understanding of its structural integrity.

Hair tensile strength was evaluated using a standardized method based on the formula where tensile strength (T) was calculated by dividing the applied force (F) by the cross-sectional area of the hair strand (A). This precise and repeatable methodology provided a quantitative baseline for assessing hair structural integrity under varying conditions.

Male and female hair loss patterns were categorized using the Norwood-Hamilton and Ludwig scales, respectively. Inter-evaluator variability was minimized through standardized training by dermatologists, with agreement among evaluators confirmed by high Fleiss Multirater Kappa values. This ensured consistency in the scoring of hair loss patterns, reinforcing the reliability of the methodology.

Collectively, these methodologies form a comprehensive framework for assessing hair and scalp health. By focusing on precision and standardization, this study provides a robust foundation for future research aimed at evaluating the efficacy of hair care products in AGA and other hair-related disorders. These methods not only enable consistent and reliable data collection but also pave the way for the development of evidence-based treatments, addressing the growing need for scientifically validated approaches in the management of hair and scalp health.

This study established standardized methods for assessing hair and scalp parameters, providing a strong foundation for future research. Expanding the sample size in subsequent studies will enhance the generalizability of these findings, ensuring broader applicability. Additionally, incorporating multiple time-point evaluations and control over dietary habits will allow for a more comprehensive understanding of AGA progression and long-term treatment efficacy.

A key limitation of this validation study is its single-center design, which may restrict the generalizability of the findings across diverse clinical settings and geographic regions. In future multicenter studies, a major challenge will be ensuring the consistent application and evaluation of these validated methods across different sites. Variability in the interpretation of assessment techniques, differences in observer expertise, and potential inconsistencies in instrument operation may introduce inter- and intra-observer variability, ultimately impacting the reliability and accuracy of the results.

To address this challenge, a preliminary validation phase could be implemented at each participating site before the initiation of a multicenter study. This could involve clinical investigators, such as dermatologists, conducting a blinded evaluation of a standardized set of 50 pre-selected images to assess scoring consistency across sites. Additionally, standardized training and calibration sessions for both evaluators and instrument operators should be conducted to minimize variability in assessment techniques and ensure uniform application of the validated methods. Such an approach would enhance the reproducibility, accuracy, and robustness of assessments, thereby strengthening the reliability of findings in future multicenter clinical studies.

Several validation studies have been conducted to assess various methodologies for evaluating hair growth parameters. This study confirms the reliability and consistency of the adherent scalp flaking score (ASFS), phototrichogram, and the 60-second hair combing test as objective tools for evaluating dandruff severity. The significant correlations observed between ASFS and phototrichogram using CASLite Nova further support their applicability in clinical settings. To ensure reproducible and accurate results, comprehensive training for evaluators and operators is essential. These findings provide a strong foundation for future validation studies and clinical assessments, contributing to the standardization of methodologies for evaluating scalp dandruff and its treatment outcomes [16].

Among these, TrichoScan® (Tricholog GmbH, Freiburg, Germany) has emerged as a valuable tool for measuring hair density and growth. While it can be applied to study the effects of drugs or laser treatments on conditions such as hypertrichosis or hirsutism, this requires a modified software algorithm, which may be beyond the scope of certain studies. Furthermore, although phototrichograms and other hair analysis methods provide critical insights into hair growth patterns and treatment efficacy, validation studies should also incorporate additional outcome measures. Specifically, integrating patient-reported outcomes, such as quality-of-life assessments, ensures a more comprehensive evaluation of treatment benefits [17].

The HAIR-Q has demonstrated both validity and reliability, making it a valuable tool for validation studies assessing patient-reported outcomes related to hair loss. It can be utilized pre- and post-treatment in adults with hair loss due to various conditions to measure patient satisfaction and the impact of treatment. However, further research is needed to evaluate additional psychometric properties that were not examined in this study, such as responsiveness and the calculation of minimally important differences. These aspects are crucial for enhancing the interpretability and clinical applicability of the HAIR-Q in both research and practice [18].

## Conclusions

This standardization and validation study underscores the importance of developing and utilizing standardized methodologies in clinical research, particularly in hair and scalp health studies. The implementation of these accurate and reproducible techniques will enhance the reliability of efficacy data for both current and future AGA treatments while establishing a benchmark for advancing research in hair restoration and scalp health. These methodologies provide a reliable and scientifically grounded approach to studying hair regrowth, paving the way for evidence-based treatments that can address the diverse needs of individuals with AGA and other hair-related disorders.
